# Stable isotopes of carbon and nitrogen help to predict the belowground communities at a regional scale

**DOI:** 10.1038/s41598-017-07517-w

**Published:** 2017-08-04

**Authors:** Bing Wang, Ying Wu, Dima Chen

**Affiliations:** 1 State Key Laboratory of Vegetation and Environmental Change, Institute of Botany, Chinese Academy of Sciences, Beijing, 100093 China; 2 College of Life Sciences, University of Chinese Academy of Sciences, Beijing, 100049 China

## Abstract

At the regional scale, although environmental factors are known to shape the distributions of belowground communities in terrestrial ecosystems, these environmental factors account for relatively low percentages of the variation in belowground communities. More of this variation might be explained by considering ecosystem stable isotopic values, which can provide insight into environmental conditions. Here, we investigated ecosystem (plant and soil) δ^13^C and δ^15^N values and belowground communities (microbes and nematodes) as well as environmental factors (climates, soils, and plants) across the Mongolian Plateau. The regression analyses showed that plant isotopic values were more closely associated with belowground communities than soil isotopic values, while ecosystem δ^13^C values were more closely associated with the belowground communities than ecosystem δ^15^N values. We also found isotopic values were more closely associated with nematode communities than microbial communities. Variation partioning analyses indicated that environmental variables together explained 16–45% of total variation in belowground communities. After isotopic variables were added as predictors to the variation partition analyses, the explanation of the variance was improved by14–24% for microbial communities and was improved by 23–44% for nematode communities. These findings indicate that isotopic values could be used to predict the properties of belowground communities at a regional scale.

## Introduction

Determinants of the spatial distribution of aboveground and belowground communities is a relevance issue of community ecology^1^. Although the spatial distributions of both aboveground^2, 3^ and belowground communities^4, 5^ have been well documented at regional scales, we still lack comprehensive understanding about the determinants of belowground communities across a broad range of environmental gradients^6^. This critical knowledge gap limit our understanding of the importance of these communities in regulating interactions in soil food webs and ecosystem process^6, 18^. A growing number of observations have demonstrated that climate^4, 5, 7^, soil variables^7, 8^, and plant characteristics^9, 10^ help shape the structure and composition of belowground communities (mostly microbial communities) at regional scale in terrestrial ecosystems. However, these environmental factors still have relatively low ability to predict the variation in belowground communities at regional scale^5, 9, 10^. In addition, these studies have not yet detected how different trophic levels of soil micro-food webs (e.g., microbes and nematodes) are regulated by these environmental variables, and this is especially true for the semi-arid grasslands on the Mongolian Plateau, which have contrasting climatic, plant, and soil conditions^5^.

Terrestrial carbon (C) and nitrogen (N) cycling is central to predicting future patterns of many ecosystem processes and functions (e.g., primary productivity, C and N sequestration, nutrient fluxes)^11, 12^. Previous studies suggested that measurement of ecosystem (plant and soil) stable isotopic compositions (δ^13^C and δ^15^N) can provide insight into soil C and N cycling^13, 14^. For example, Garten *et al*.^15^ and Craine *et al*.^16^ documented that plant δ^15^N values increased with increasing N availability and potential N mineralization across natural N supply or N availability gradients. Furthermore, it has been well documented that soil C and N cycling are also greatly affected by belowground communities through respiration and N mineralization^17, 18^, and understanding the belowground communities that drive these processes has long been a goal of soil scientists^19, 20^. For example, a recent regional-scale study showed that microbial biomass alone predicted 61% of the variance in C mineralization rates at 0–5 cm depth in mineral soils^21^. Overall, both the ecosystem stable isotopic compositions and belowground communities have great effects on the soil C and N cycling hinted that there properly exist an inherent correlation between them, and therefore our understanding of belowground communities could be increased by considering ecosystem stable isotopic compositions. Stable isotope information can provide insight into climate conditions and changes in the plant community composition, water-use efficiency, nitrogen status, and terrestrial ecosystem functions along environmental gradients^13, 14, 22^. Information on isotopic compositions could be useful in evaluating spatial patterns of belowground communities and their feedback to global change ecology^15, 23^. For example, one report has provided direct evidence regarding the relationship between isotopic values and belowground communities^24^; that report documented a positive correlation between the δ^15^N values and abundance of ammonia oxidizing archaea at four sites in Arizona and in Hawai’i. However, it is still unclear whether ecosystem δ^13^C and δ^15^N values can be used to predict the spatial patterns in belowground communities along environmental gradients at a regional scale.

**Figure 1:**
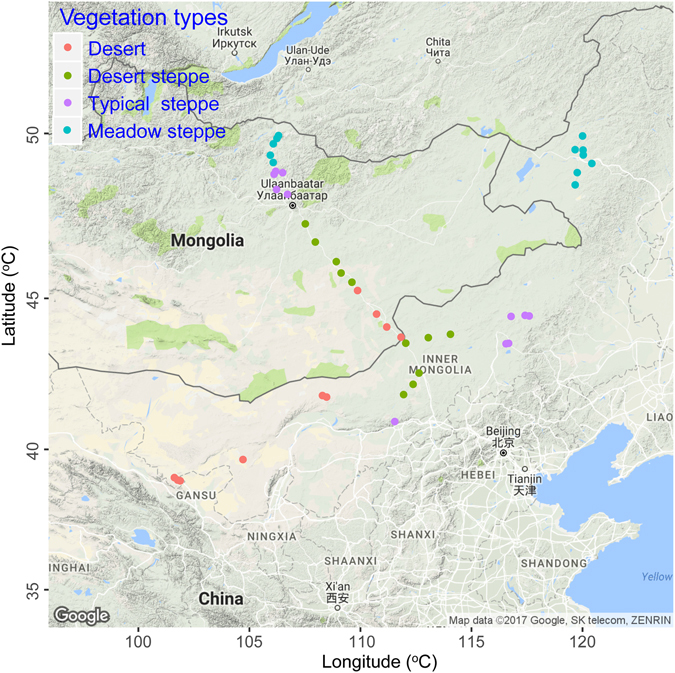
Locations of the study sites along the east–west (Inner Mongolia) and north–south (Mongolia) transects across the Mongolian Plateau^5, 26^. The map was created by the Google Maps module in R (https://cran.r-project.org/package=ggmap).

The natural semi-arid grassland ecosystem on the Mongolia Plateau covers about 10% of the global land surface, and even though this plateau stores substantial quantities of C and N^25^, its belowground communities have seldom been studied^5, 26^. In the present study, we assessed the relationships between ecosystem (plant and soil) isotopic values (δ^13^C and δ^15^N) and changes in belowground communities along environmental gradients (climate, soils, and plants) on two perpendicular transects on the Mongolia Plateau (Fig. 1). We attempted to answer three questions. First, how do the ecosystem isotopic values relate to the soil microbial and nematode communities at the regional scale? Second, after controlling for environmental variables, how much of the variance in the functional groups of the belowground microbial and nematode communities is explained by isotopic values? Third, what explains the relationships between the ecosystem isotopic values and soil microbial and nematode communities on the Mongolia Plateau?

Specifically, we tested three hypotheses. H1: We hypothesize that belowground communities on the Mongolia Plateau will be more closely associated with plant isotopic values than with soil isotopic values and will be more closely associated with ecosystem δ^15^C values than with ecosystem δ^13^N values. Because photosynthates released by roots are a major source of available C and N for belowground communities^27, 28^. In addition, the availability of C is a better predictor of belowground communities than the availability of N^7, 29^. H2: We hypothesize that the isotopic values will be more closely associated with soil microbes than with soil nematodes along the environmental gradients. Because bottom-up effects derived from C and N substrates are often depend on trophic levels in micro-food web, with stronger effects on lower trophic levels (e.g., soil microbes) than on higher trophic levels (e.g., soil nematodes)^30–32^. H3: We hypothesize that, after considering ecosystem stable isotopic values, the explanation of the variance in belowground communities will be greatly improved than the situations which only consider environmental variables. Because stable isotope information can provide insight not only into environmental variables but also into water-use efficiency and terrestrial ecosystem functions along environmental gradients^13, 14, 22^.

## Results

### Relationships between isotopic values and belowground communities

From desert to meadow steppe, we found that MAP, SOC, TSN, TSP, plant C content increased while MAT and plant N content decreased (Table S1). Soil δ^13^C decreased from −22.2 to −25.3‰, and plant δ^13^C decreased from −18.8 to −25.9‰ (Table 1). Similarly, soil δ^15^N decreased from 6.62 to 4.77‰, and plant δ^15^N decreased from 3.19 to 0.26‰ (Table 1). For microbes, total soil microbial biomass, biomass of all microbial groups, B/F ratio increased while microbial community structure decreased from desert to meadow steppe (Table 1). For nematodes, total nematode abundance, abundance of four nematode trophic groups, nematode taxon richness, and nematode community structure increased from desert to meadow steppe (Table 1).

**Table 1:** Characteristics of ecosystem isotopic values, soil microbes, and nematodes of the four vegetation types in the Mongolian grassland.

**Characteristics**	**Desert**	**Desert steppe**	**Typical steppe**	**Meadow steppe**
**Ecosystem isotopic values**				
Plant δ^13^C (‰)	−18.8 (0.4)^c^	−24.4 (0.4)^b^	−25.8 (0.1)^a^	−25.9 (0.2)^a^
Soil δ^13^C (‰)	−22.2 (0.2)^d^	−22.8 (0.2)^c^	−24.5 (0.1)^b^	−25.3 (0.1)^a^
Plant δ^15^N (‰)	3.19 (0.24)^c^	2.12 (0.18)^b^	0.48 (0.12)^a^	0.26 (0.09)^a^
Soil δ^15^N (‰)	6.62 (0.29)^b^	6.81 (0.20)^b^	4.95 (0.14)^a^	4.77 (0.12)^a^
**Microbial community**				
Total FAs (nmol g^−1^)	9.64 (0.82)^a^	18.77 (0.90)^b^	24.71 (1.67)^c^	29.22 (2.08)^c^
Ba FAs (nmol g^−1^)	5.33 (0.46)^a^	10.01 (0.48)^b^	12.97 (0.88)^c^	15.57 (1.12)^c^
Fu FAs (nmol g^−1^)	0.45 (0.04)^a^	0.54 (0.03)^ab^	0.61 (0.04)^bc^	0.72 (0.05)^c^
Act FAs (nmol g^−1^)	1.21 (0.13)^a^	2.52 (0.17)^b^	2.61 (0.16)^b^	5.77 (0.31)^c^
AMF FAs (nmol g^−1^)	1.91 (0.15)^a^	4.13 (0.22)^b^	4.05 (0.27)^b^	8.37 (0.51)^c^
B/F	12.83 (0.45)^a^	19.37 (0.94)^b^	21.62 (0.79)^bc^	22.76 (0.74)^c^
MCS	0.80 (0.16)^d^	−0.05 (0.14)^c^	−0.32 (0.05)^b^	−0.45 (0.07)^a^
**Nematode community**				
TNA (Ind. 100 g^−1^)	106 (13)^a^	294 (30)^b^	509 (35)^c^	598 (23)^d^
BF (Ind. 100 g^−1^)	76 (9)^a^	173 (16)^b^	283 (23)^c^	346 (17)^d^
FF (Ind. 10 g^−1^)	18 (3)^a^	68 (8)^b^	99 (9)^c^	107 (7)^c^
PF (Ind. 100 g^−1^)	4.1 (0.7)^a^	22.8 (4)^b^	64.6 (4.7)^c^	60.5 (4.6)^d^
OC (Ind. 100 g^−1^)	8.1 (1.9)^a^	30.2 (5.1)^b^	61.6 (6.7)^c^	84.8 (6.7)^c^
NTR	8.1 (0.7)^**a**^	12.7 (0.5)^**b**^	16.8 (0.4)^**c**^	16.5 (0.4)^**c**^
NCS	−1.06 (0.10)^a^	−0.05 (0.10)^b^	0.40 (0.12)^c^	0.75 (0.09)^d^

Values are means (SE). Different letters in a row indicate significant differences among the four vegetation types (one-way ANOVA, *P* < 0.05). Microbial community: FAs, phospholipid fatty acid; Ba FAs, bacterial FAs; Fu FAs, fungal FAs; Act FAs, actinobacterial FAs; AMF FAs, arbuscular mycorrhizal fungal FAs; B/F, ratio of bacterial FAs to fungal FAs; MCS, microbial community structure; Nematode community: TNA, total nematode abundance; BF, bacterial-feeding nematodes; FF, fungal-feeding nematodes; PF, plant-feeding nematodes; OC, omnivore + carnivore nematodes; NTR, nematode taxon richness; NCS, nematode community structure.

**Figure 2:**
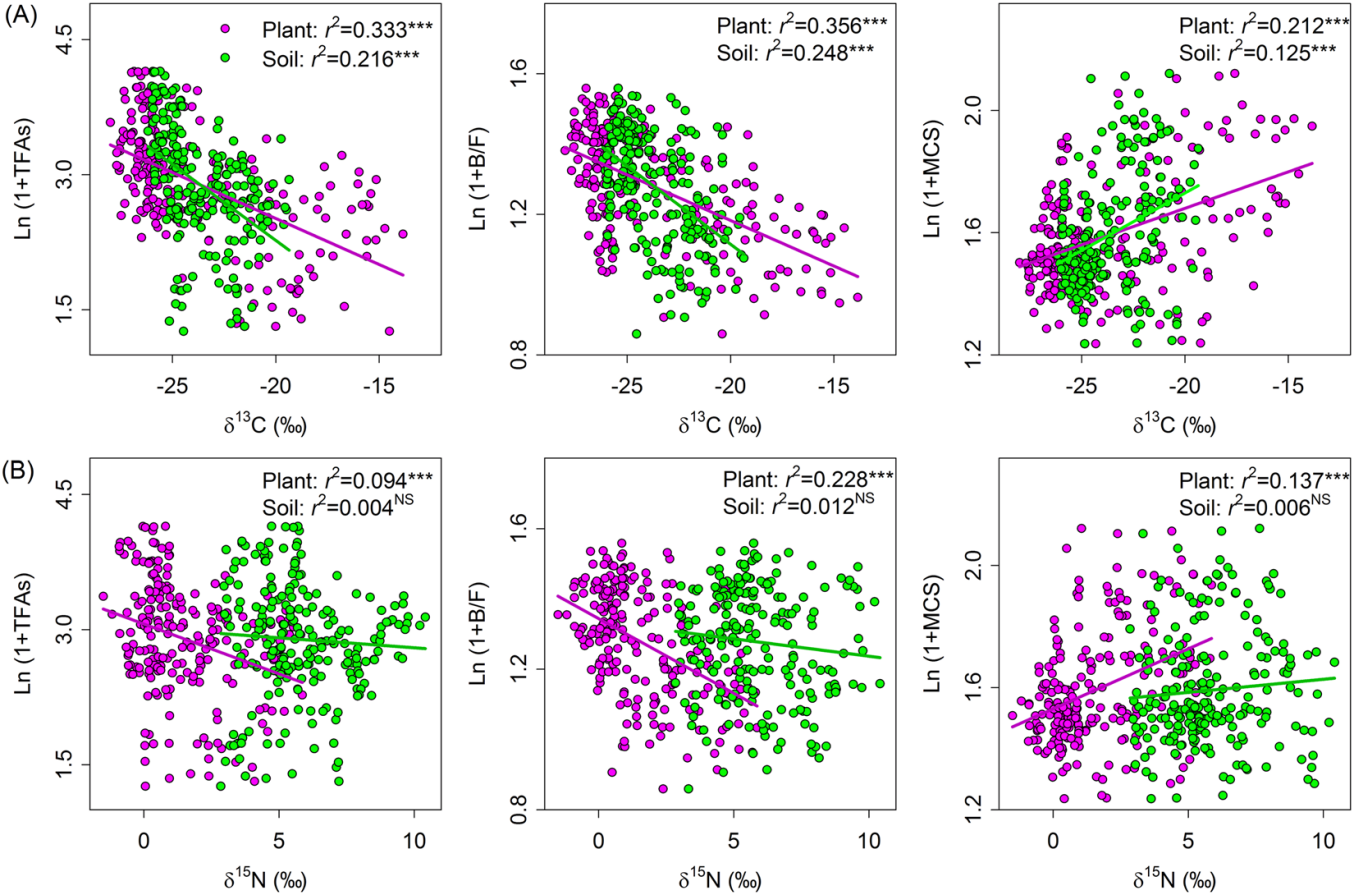
Relationships between microbial community variables and (**A**) δ^13^C isotopic values and (**B**) δ^15^N isotopic values of plants (pink symbols) and soils (green symbols) at the regional scale in the Mongolian grassland. Abbreviations are explained in Table 1. Regression analysis is indicated by *r* ^*2*^ and significance level (NS, *P* > 0.05; **P < 0.01; ***P < 0.001).

**Figure 3:**
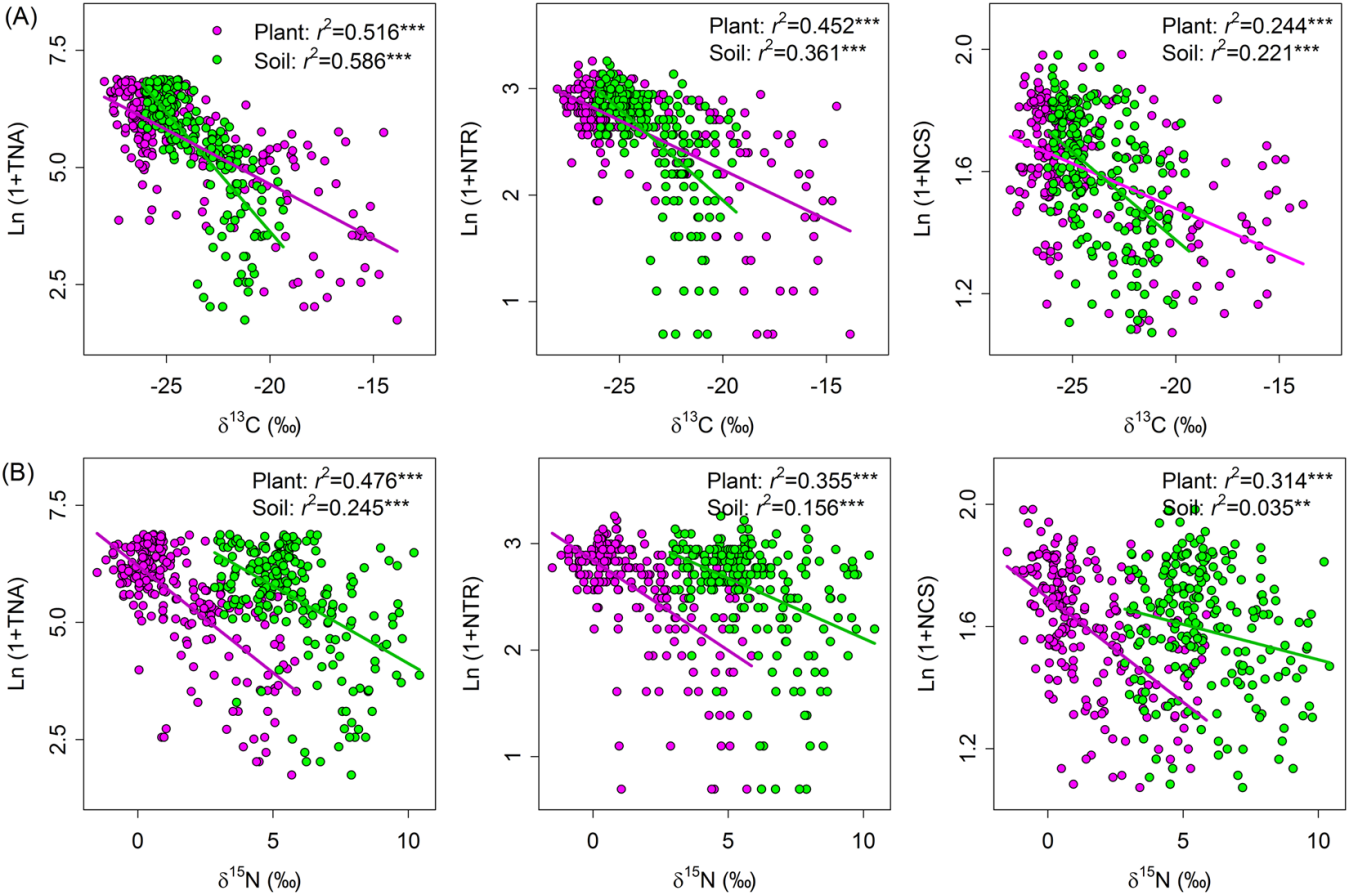
Relationships between nematode community variables and (**A**) δ^13^C isotopic values and (**B**) δ^15^N isotopic values of plants (pink symbols) and soils (green symbols) at the regional scale in the Mongolian grassland. Abbreviations are explained in Table 1. Regression analysis is indicated by *r* ^*2*^ and significance level (**P < 0.01; ***P < 0.001).

**Figure 4:**
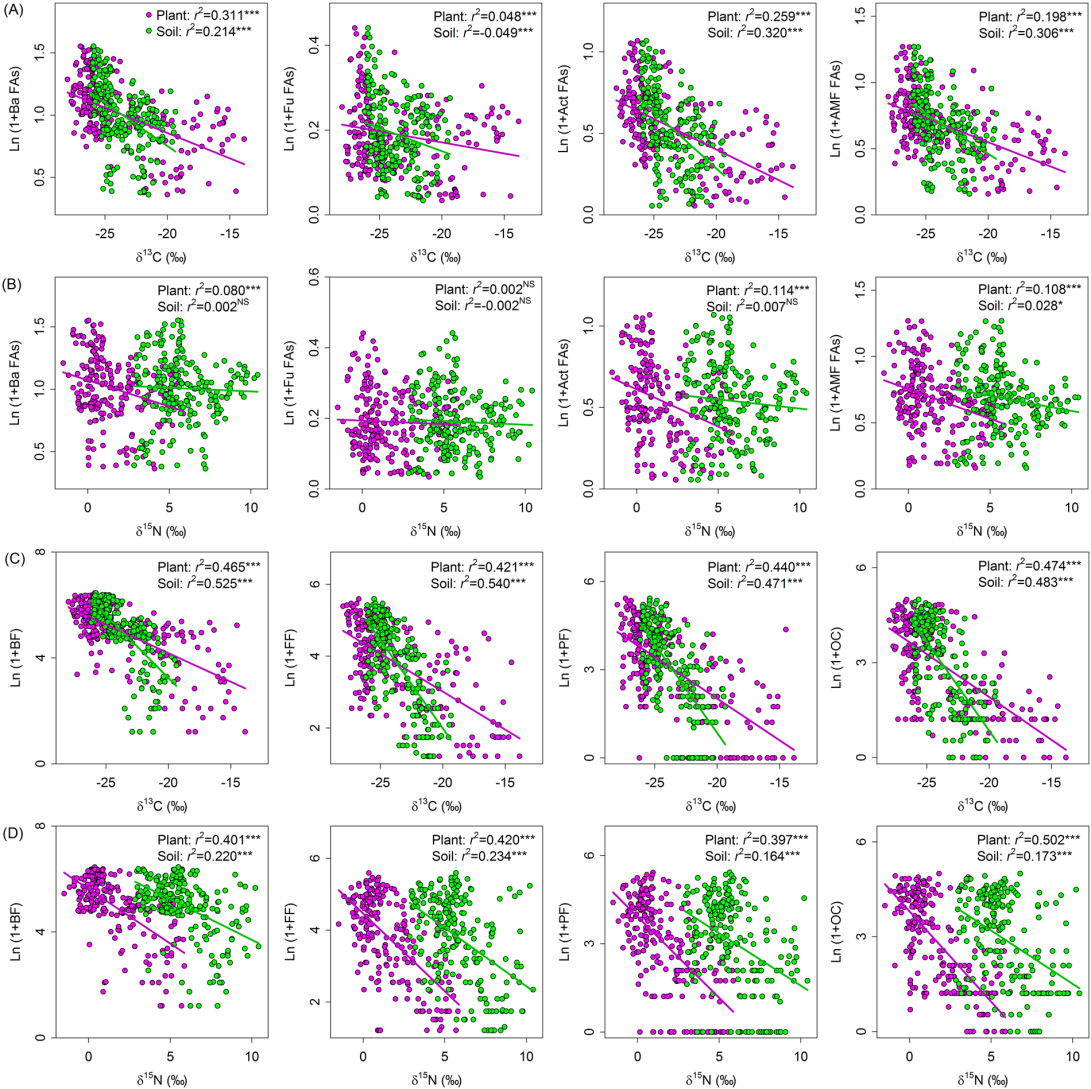
Relationships between additional microbial and nematode community variables and (**A** and **C**) δ^13^C isotopic compositions and (**B** and **D**) δ^15^N isotopic compositions of plants (pink symbols) and soils (green symbols) at the regional scale in the Mongolian grassland. Abbreviations are explained in Table 1. Regression analysis is indicated by *r* ^2^ and significance level (NS, *P* > 0.05; *P < 0.05; **P < 0.01; ***P < 0.001).

Regression analyses showed that ecosystem isotopic values were closely associated with most variables in belowground communities (Figs 2–4). Ecosystem δ^13^C values (plant and soil) were negatively related to total microbial biomass, the biomass of all four microbial groups, and B/F ratios, and were positively related to microbial community structure across the Mongolian Plateau (Figs 2A and 4A). Plant δ^15^N values were negatively related to total microbial biomass, the biomass of all four microbial groups, and B/F ratios while were positively related to microbial community structure. Soil δ^15^N values, however, were not related to any of the microbial variables (Figs 2B and 4B). Ecosystem δ^13^C or ^15^N values were negatively associated with total nematode abundance, the abundance of all nematode trophic groups, nematode taxon richness, and nematode community structure (Figs 3 and 4C). Soil nematode variables were more closely associated with ecosystem δ^13^C values than with ecosystem ^15^N values (Figs 3 and 4D).

### Relative effects of isotopic values and environmental variables on belowground communities

**Figure 5:**
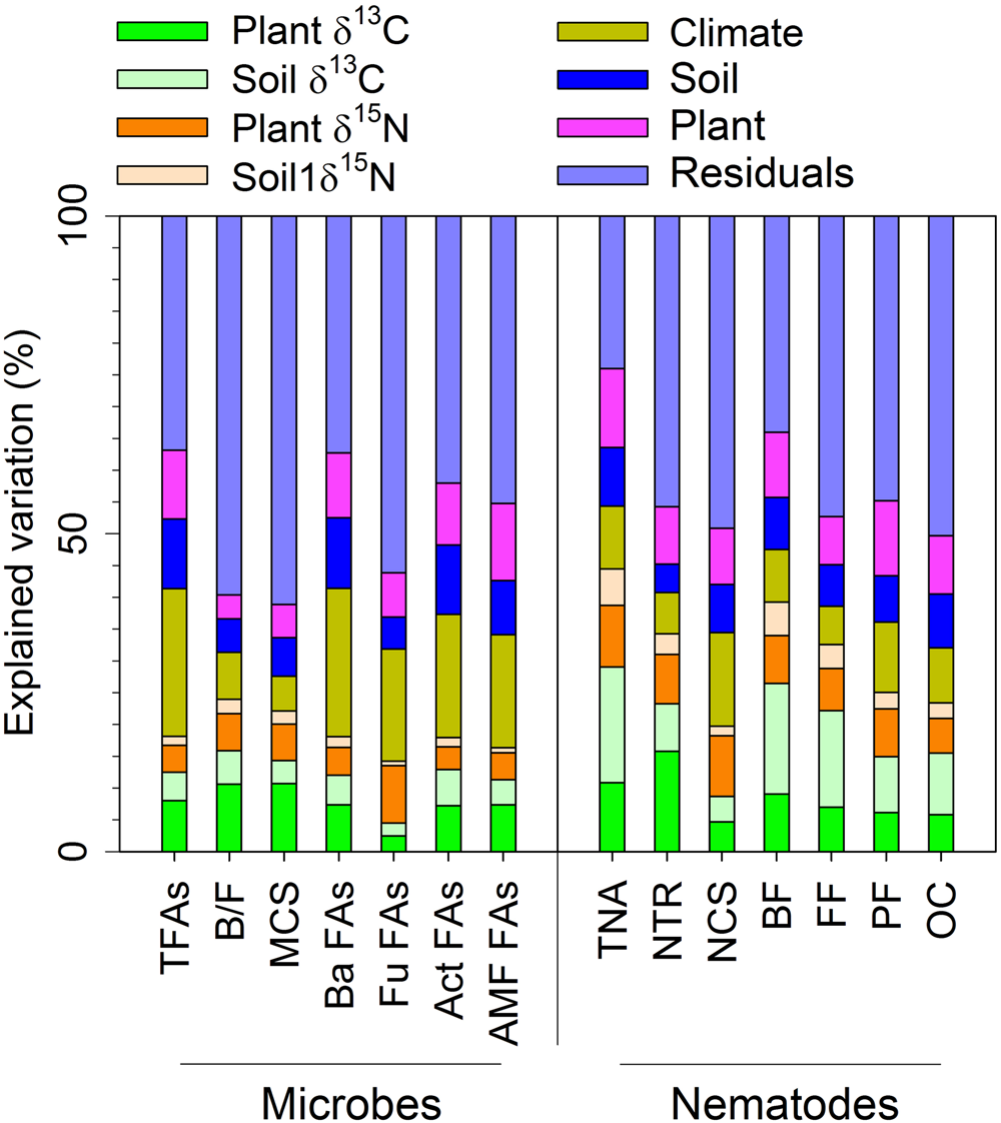
Percentages of variation in soil microbial and nematode properties explained by ecosystem isotopic values (δ^13^C and δ^15^N) at the regional scale in grasslands on the Mongolian Plateau. Abbreviations are explained in Table 1.

Variation partition analyses showed that environmental variables (climate, plants, and soils) together explained 16–45% of total variation in in microbial communities and 20–32% of total variation in in nematode communities (Fig. 5). When considering the ecosystem isotopic variables, we found that ecosystem δ^13^C values explained 5–16% of the total variation in microbial communities and 9–29% of the total variation in nematode communities, while ecosystem δ^15^N values explained 5–10% of the total variation in microbial communities and 8–15% of the total variation in nematode communities (Fig. 5). Plant isotopic values explained 11–16% of the total variation in microbial communities and 11–23% of the total variation in nematode communities, while soil isotopic values explained only 3–7% of the total variation in microbial communities and 5–24% of the total variation in nematode communities (Fig. 5). Overall, our results indicated that, after ecosystem isotopic variables were added as predictors to the variation partition analyses, the explanation of the variance was improved by14–24% for microbial communities and was improved by 23–44% for nematode communities (Fig. 5). Partial regressions further indicated that, after environmental variables were controlled for, most variables in nematodes significantly related with ecosystem isotopic values. For microbes, most variables significantly related with plant δ^13^C values while did not relate with soil δ^15^N values; part of microbial variables related with soil δ^13^C values or plant δ^15^N values (Table 2).

**Table 2:** Partial correlations between ecosystem isotopic values and belowground communities when controlling for environmental variables at the regional scale in the Mongolian grassland (n = 220).

	Plant δ^13^C	Soil δ^13^C	Plant δ^15^N	Soil δ^15^N
**Microbial community**				
Total FAs	0.324***	0.201**	−0.020^NS^	0.097^NS^
Ba FAs	0.338***	0.210**	0.008^NS^	0.073^NS^
Fu FAs	0.328***	0.061^NS^	0.153*	0.043^NS^
Act FAs	0.237***	0.153*	−0.024^NS^	0.000^NS^
AMF FAs	0.283***	0.082^NS^	−0.046^NS^	0.119^NS^
B/F	−0.177**	0.076^NS^	−0.276***	−0.076^NS^
MCS	0.123*	−0.079^NS^	0.222***	0.005^NS^
**Nematode community**				
TNA	−0.330***	−0.530***	−0.292***	−0.366***
BF	−0.268***	−0.479***	−0.216***	−0.330***
FF	−0.219***	−0.409***	−0.215***	−0.251***
PF	−0.103^NS^	−0.227***	−0.113^NS^	−0.165*
OC	−0.087^NS^	−0.201**	−0.162*	−0.121*
NTR	−0.435***	−0.243***	−0.224***	−0.158*
NCS	0.042^NS^	0.049^NS^	−0.231***	0.051^NS^

Correlation analysis is indicated by *r* and significance level (NS, *P* > 0.05; **P < 0.01; ***P < 0.001).

## Discussion

### Linkages of ecosystem isotopic values to belowground communities

Our regional-scale study revealed that belowground communities were more closely associated with plant isotopic values than with soil isotopic values, which was consistent with our first hypothesis. As primary producers and sources of resources/energy for soil organisms in the decomposer food web, plant-related variables can highly associated with composition and structure of belowground communities and functioning they regulated^27, 28, 33^. Therefore, the isotopic values of plants will greatly affect the isotopic values of belowground communities^28, 34^. Consistent with our first hypothesis, belowground communities were more closely associated with ecosystem δ^13^C values than with ecosystem δ^15^N values. The higher percentages of variation in belowground communities explained by ecosystem δ^13^C values than ecosystem δ^15^N values has several explanations. First, C availability is a better predictor of belowground communities than N availability because C provides the energy required by most soil organisms^7, 29^. Second, plant δ^13^C values reflect plant intrinsic characteristics, plant genetic types and life forms by controlling photosynthetic capacity^35, 36^. Third, in contrast to δ^13^C isotope discrimination, N isotope discrimination is far from complete because the isotopic composition of N leaving the system (e.g., volatilization, mineralization and leaching, and plant uptake) is the most relevant determinant of ecosystem δ^15^N values^23, 37^.

Inconsistent with our second hypothesis, ecosystem isotopic values explained less of the variation in microbes than in nematodes. Bottom-up effects on lower trophic levels (e.g., soil microbes) are generally stronger than on higher trophic levels (e.g., soil nematodes)^30, 31^. In the present study, however, ecosystem isotopic values explained more of the variation in nematode communities than in microbial communities. This unexpected finding might be explained by that nematode survival and therefore abundance is very dependent on soil moisture than microbial communities^5^. Previous regional-scale reports documented that ecosystem isotopic values are strongly associated with climate and especially with precipitation^37–39^. Consistent with these previous reports, our results indicated strong negative relationships between ecosystem δ^13^C and δ^15^N values and MAP on the Mongolia Plateau. The close relationship between ecosystem isotopic values and precipitation could help explain why nematode communities were more sensitive to ecosystem isotopic values than microbial communities^5, 40^.

### Potential mechanisms explaining the linkages between isotopic values and belowground communities

Our results showed that ecosystem isotopic values were strongly associated with environmental factors (climate, soil, and plant) (Table S4). Many previous studies have documented that the variation in ecosystem isotopic values is closely associated with climate, soils, and plants^37–39^. Regional-scale studies have indicated that climate and especially precipitation is predominant in determining ecosystem δ^13^C or δ^15^N values^37, 39^. Also, our finding that ecosystem δ^13^C or δ^15^N values increased with increasing MAT is consistent with finding from Männel *et al*.^41^, which documented a clear and positive relationship between plant δ^13^C values and MAT in arid and semiarid grasslands. Ecosystem δ^13^C or δ^15^N values were related to plant characteristics in the current study, which again can be explained by the effects of these characteristics on photosynthetic capacity^35, 36^. In addition, the negative relationships between soil factors and soil δ^13^C or δ^15^N values in the current study (Table S4) were consistent with a previous study in alpine grasslands^42^, which showed that soil clay content and soil C/N explained much of the variation in soil δ^15^N values. Overall, the results indicate that regional-scale isotopic composition can be a good predictor of climate, plants, and soils.

Consistent with our third hypothesis, our variation partitioning analyses showed that, besides environmental variables, ecosystem isotopic values independently explained much of the spatial variation in belowground communities across the Mongolia Plateau. Specifically, after isotopic variables were added as predictors to the variation partition analyses, the explanation of the variance was improved by14–24% for microbial communities and was improved by 23–44% for nematode communities. Our study, to our knowledge, is the first one to provide direct information concerning the linkages between ecosystem isotopic values and more than one trophic level of belowground communities at a regional scale. Our findings suggest that ecosystem isotopic values could be used to predict the characteristics of belowground communities at a regional scale. Because ecosystem δ^13^C and δ^15^N values are related to C assimilation and to N status as influenced by water stress and cycling of elements, ecosystem isotopic values are closely coupled to belowground communities^43, 44^. The relationships between isotopic composition and below-ground communities have seldom been separated from environmental factors in previous studies, which may be one reason why isotopic composition explained only low percentages of the total variation in belowground communities in these studies^5, 9, 10^. To assess the potential of ecosystem soils to sequester C and N, researchers must be able to readily evaluate belowground communities^6, 18^. Interestingly, the ecosystem isotopic composition explained similar percentage of the total variation in specific variable in microbial or nematode communities. This could be due to the fact that their similar responses to changes in ecosystem isotopic values across the Mongolia Plateau. Although we were not able to identify detailed mechanisms that how ecosystem isotopic values shaped the belowground communities in detail, our results from the Mongolian Plateau indicate that ecosystem isotopic values help to predict the spatial variation in belowground communities for semi-arid grasslands.

### Implications of ecosystem isotopic values for semi-arid grasslands

Numerous studies have concluded that stable C and N isotopic signatures provide insight into the biotic and abiotic factors controlling ecosystem functions (e.g., the cycling of C and N)^13, 45^. Because the community-averaged foliar δ^13^C value is related to C assimilation and to the diffusion of CO_2_ as influenced by water stress and cycling of elements, and adaptations of plants to local climate therefore lead to plant δ^13^C values that are closely coupled to N and C cycling^43, 46^. As part of the largest contiguous grassland in the world, the Mongolian Plateau is an important terrestrial ecosystem that greatly affects global C and N storage and cycling^47^. Therefore, understanding the mechanisms governing belowground communities is crucial for evaluating ecosystem C and N balance in the Mongolia Plateau and its feedbacks to climate change. In recent decades, the semi-arid grassland ecosystems on the Mongolia Plateau have been affected by multiple anthropogenic stressors (e.g., global climate change and land-use change). A significant consequence of these anthropogenic stressors is the widespread changes in both belowground communities and ecosystem functions^48^. To rapidly assess the potential of ecosystem soils to sequester C and N, researchers must be able to readily evaluate belowground communities^13, 14^. The assessment of ecosystem stable isotopes of C and N allows researchers to rapidly and precisely measure belowground communities and hence estimate C or N storage and cycling. Our results from the Mongolian Plateau indicate that predictive models of C and N sequestration regulated by belowground communities should include assessment of ecosystem isotopic values.

## Materials and Methods

### Study area and sampling design

The research location as well as sampling design were explained thoroughly by Chen *et al*.^5, 26^. In brief, we developed two vertical regional-scale transects on the Mongolian Plateau in the Eurasian steppe in August 2010 and 2011, respectively (Fig. 1). Both transects went across four major vegetation types, consisting of meadow steppe, typical steppe, desert steppe, and desert. From desert to meadow steppe, the soil type of each vegetation type was dominated by light brown, calcic brown, typical chestnut, and dark chestnut, respectively. About 10–12 sites were selected for each vegetation type (A total of 44 sites); these sites were not foraged obviously by large mammals. For each site, the mean annual precipitation (MAP) and mean annual temperature (MAT) during 1970–2000 were obtained from the WorldClim database (http://www.worldclim.org/current)^49^.

### Sampling and measurements

At each site, aboveground net primary productivity (ANPP) was determined in five 1 × 1 m herbaceous subplots or 5 × 5 m shrub subplots situated randomly within a 100 × 100 m site. All plant materials in each subplot were oven-dried at 65 °C for 48 h and weighed as ANPP. We classified all plant species into five plant functional groups based on life forms^50^: perennial rhizome grasses, perennial bunchgrasses, perennial forbs, shrubs and semi-shrubs, and annuals. Principal component analysis (PCA), based on the relative biomass proportion (%) of the five plant functional groups, was performed, and the first ordination axes (PC1) was used as indicator of plant community structure (Table S2). Then, all of the aboveground live plant materials from each subplot were ground with a ball mill. These ground plant samples were used to estimate plant C and N contents and δ^13^C and δ^15^N values. Soils from 0 to 20 cm depth were randomly collected by taking three soil cores (7-cm-diameter) in each subplots. The soils from the three cores were mixed *in situ* to form one composite soil sample. After passed through 2-mm-mesh sieves, the soil was separated into two parts. One part was maintained fresh for determination of soil moisture, soil microbes, and soil nematodes. The other part, which was air-dried, was used to determine soil δ^13^C and δ^15^N values, soil pH, soil organic C content (SOC), total soil N content (TSN), and total soil phosphorus (TSP). All results are expressed on a dry weight basis.

### Plant and soil δ^13^C and δ^15^N values

The δ^13^C and δ^15^N values of plant and soil samples were determined with an isotope ratio mass spectrometer (Delta Plus XP, Thermo Finnigan, Berlin, Germany) coupled with an elemental analyser (Euro EA 3000, EuroVector, Milan, Italy). The C or N isotope data were specified as δ^13^C or δ^15^N relative to the Vienna Pee Dee Belemnite standard: δ^13^C or δ^15^N (‰) = (*R*_sample_/*R*_standard_ − 1) × 1000, where *R*_sample_ and *R*_standard_ are the ratios of ^13^C/^12^C or ^15^N/^14^N in the sample and standard.

### Soil microbial and nematode communities

Composition and structure of soil microbial communities were analyzed by phospholipid fatty acid (PLFA) technique^51^. The separated fatty acid were detected by an Agilent 6890 gas chromatograph (Agilent Technologies, Palo Alto, CA) and equipped with a MIDI Sherlock Microbial Identification System (MIDI Inc., Newark, DE, USA). The biomass of each specific FAs for each soil sample was expressed as nmol of fatty acid g^−1^ dry soil. FAs specific to bacteria, fungi, actinobacteria, and arbuscular mycorrhizal fungi were used to determine the biomass of these microbial groups^5, 26^ and to calculate fungi/bacteria ratios (F/B)^52^. Like plant community structure, microbial community structure was indicated using the first ordination axes of the relative abundances of four microbial groups (mol%) (Table S2).

Soil nematodes from 50 g of moist soil per soil sample were extracted using Baermann funnel method for 48 h. After fixation in a 4% formalin solution, the nematodes were counted with an inverted microscope. Based on morphology, the first 100 individuals were identified to genus and assigned to five trophic groups^53^: plant-feeding, bacterial-feeding, fungal-feeding, omnivorous, and carnivorous. The results of nematode abundance are expressed on 100 g dry weight basis. The number of genera was used as an indicator of taxon richness (NTR). Nematode community structure was indicated using the first ordination axes of the relative abundances of four nematode trophic groups (%) (Table S2).

### Statistical analyses

All statistical analyses were done with R 3.3.2 (R Development Core Team 2016). First, one-way ANOVAs with Tukey’s multiple range tests was performed across all variables to compare the means among vegetation types. To satisfy assumption of ANOVAs, the data for some variables were transformed to natural logarithms. Second, the relationships between ecosystem (plant and soil) isotopic values (δ^13^C and δ^15^N) and belowground communities (microbes and nematodes) were tested using linear regressions. Third, hierarchical variation partitioning analyses (with the calc.relimp function by the LMG methods in the “relaimpo” package) were used to determine the relative importance of ecosystem isotopic values and environmental factors (climate, plants, and soils) on belowground community variables^54^. LMG assigns each variable the average increase in R^2^ when it is added to a regression model containing a subset of other variables. Finally, partial regressions were used to test the relationships of partial residuals between ecosystem isotopic values and belowground community variables after the three environmental variables were controlled for. To facilitate our interpretations, we classified the environmental factors into three groups^5, 26^: (1) climate (MAP and MAT); (2) soils (pH, SOC, TSN, and TSP), and plants (ANPP, plant species richness, plant C and N contents, and plant community structure). We conducted PCA on the multiple variables for each environmental group and used the PC1 scores as indicators of each environmental group^26^. The first ordination axes (PC1) explained 54–90% of the total variance for each environmental group and was used in our analysis and interpretations (Table S3).

## Electronic supplementary material

Supplementary Information
