# Supplementary material for: Stable isotopes of carbon and nitrogen help to predict the belowground communities at a regional scale
Source: Sci Rep. 2017 Aug 4;7:7276. doi: 10.1038/s41598-017-07517-w (PMC5544679; doi:10.1038/s41598-017-07517-w)
Supplement: Supplementary file 1 — Supplementary Information [file 41598_2017_7517_MOESM1_ESM.doc]

**Supporting Information**

Stable isotopes of carbon and nitrogen help to predict the belowground communities at a regional scale

Bing Wang^1,2#^, Ying Wu^1,2#^, Dima Chen^1^*

^1^State Key Laboratory of Vegetation and Environmental Change, Institute of Botany, Chinese Academy of Sciences, Beijing, 100093, China

^2^College of Life Sciences, University of Chinese Academy of Sciences, Beijing 100049, China

^#^ These authors contributed equally to this work.

*Corresponding author: Dima Chen

State Key Laboratory of Vegetation and Environmental Change, Institute of Botany, the Chinese Academy of Sciences, 20 Nanxincun, Xiangshan 100093, Beijing, P.R. China

E-mail: [chendima@ibcas.ac.cn](mailto:chendima@ibcas.ac.cn); Tel: (+86)-10-6283-6592; Fax: (+86)-10-8259-5771

**Table S1** Characteristics of climate, soils, plants of the four vegetation types in the Mongolian grassland. Values are means (SE). Different letters in a row indicate significant differences among the four vegetation types (one-way ANOVA, *P*<0.05).

| **Characteristic** | **Desert** | **Desert steppe** | **Typical steppe** | **Meadow steppe** |
| --- | --- | --- | --- | --- |
| **Climate** |  |  |  |  |
| Mean annual precipitation (mm) | 127(1)^a^ | 213(4)^b^ | 305(6)^c^ | 359(5)^d^ |
| Mean annual temperature (^o^C) | 4.98(0.30)^d^ | 1.79(0.31)^c^ | 0.48(0.17)^b^ | -1.40(0.08)^a^ |
| **Soils** |  |  |  |  |
| Soil pH | 8.3(0.1)^c^ | 7.9(0.1)^b^ | 7.6(0.1)^b^ | 7.0(0.1)^a^ |
| Soil organic carbon (g kg^-1^) | 2.8(0.2)^a^ | 10.4(0.9)^b^ | 17.6(0.9)^c^ | 32.1(1.7)^d^ |
| Total soil nitrogen (g kg^-1^) | 0.21(0.02)^a^ | 1.05(0.09)^b^ | 1.69(0.09)^c^ | 2.75(0.14)^d^ |
| Total soil phosphorus (g kg^-1^) | 0.22 (0.01)**^a^** | 0.38 (0.03)**^b^** | 0.39 (0.03)**^b^** | 0.54 (0.02)**^c^** |
| **Plants** |  |  |  |  |
| ANPP (g m^-2^) | 29(3)^a^ | 88(7)^b^ | 176(9)^c^ | 215(11)^d^ |
| Plant species richness | 7.7(0.3)^a^ | 11.5(1.0)^b^ | 11.4(0.6)^c^ | 20.3(1.1)^d^ |
| Plant community carbon (%) | 41.0(0.3)^a^ | 43.6(0.3)^b^ | 46.2(0.2)^c^ | 46.8(0.2)^c^ |
| Plant community nitrogen (%) | 1.94(0.04)^b^ | 2.12(0.06)^b^ | 1.61(0.03)^a^ | 1.66(0.02)^a^ |
| Plant community structure | 1.09(0.09)^d^ | 0.13(0.12)^c^ | -0.34(0.10)^b^ | -0.71(0.08)^a^ |

**Table S2** Results of principal component analysis (PCA) of plant, microbial, and nematode community structure at the regional scale in grasslands on the Mongolian Plateau.

| **Variables** | **PC1** |
| --- | --- |
| **Plant community structure** |  |
| Annuals/biannuals (%) | 0.537 |
| Perennial bunchgrasses (%) | -0.883 |
| Perennial forbs (%) | 0.478 |
| Perennial rhizome grass (%) | -0.336 |
| Shrubs and semi-shrubs (%) | 0.411 |
| **Cumulative (%)** | 42 |
| **Microbial community structure** |  |
| Bacterial PLFAs (mol%) | 0.382 |
| Fungal PLFAs (mol%) | 0.888 |
| Actinobacterial PLFAs (mol%) | -0.867 |
| Arbuscular mycorrhizal fungal PLFAs (mol%) | 0.259 |
| **Cumulative (%)** | 44 |
| **Nematode** **community structure** |  |
| Bacterial-feeding nematodes (%) | -0.982 |
| Fungal-feeding nematodes (%) | 0.566 |
| Plant-feeding nematodes (%) | 0.709 |
| Omnivore+carnivore nematodes (%) | 0.556 |
| **Cumulative (%)** | 53 |

**Table S3** Results of principal component analysis (PCA) of climate, soils, and plants at the regional scale in grasslands on the Mongolian Plateau.

| **Variable** | **PC1** |
| --- | --- |
| **Climate** |  |
|  |  |
| Mean annual precipitation (mm) | 0.953 |
| Mean annual temperature (^o^C) | -0.953 |
| **Cumulative (%)** | 90 |
| **Soils** |  |
| Soil pH | -0.803 |
| Soil organic carbon (g kg^-1^) | 0.941 |
| Total soil nitrogen (g kg^-1^) | 0.946 |
| Total soil phosphorus (g kg^-1^) | 0.83 |
| **Cumulative (%)** | 78 |
| **Plants** |  |
| Aboveground net primary productivity (g m^-2^) | 0.83 |
| Plant species richness | 0.646 |
| Plant community carbon (%) | 0.812 |
| Plant community nitrogen (%) | -0.578 |
| Plant community structure | -0.776 |
| **Cumulative (%)** | 54 |

**Table S4** Pearson correlations between ecosystem isotopic values and in climate, soil, and plant variables at the regional scale in the Mongolian grassland (n=220). Correlation analysis is indicated by *r* and significance level (NS, *P*> 0.05; **, *P*< 0.01; ***, *P*< 0.001).

| **Variables** | Plant δ^13^C (‰) | Soil δ^13^C (‰) | Plant δ^15^N (‰) | Soil δ^15^N (‰) |
| --- | --- | --- | --- | --- |
| **Climate** |  |  |  |  |
|  |  |  |  |  |
| Mean annual precipitation (mm) | -0.726*** | -0.767*** | -0.696*** | -0.463*** |
| Mean annual temperature (^o^C) | 0.706*** | 0.620*** | 0.531*** | 0.101^NS^ |
| **Soils** |  |  |  |  |
| Soil pH | 0.528*** | 0.546*** | 0.335*** | -0.043^NS^ |
| Soil organic carbon (g kg^-1^) | -0.647*** | -0.743*** | -0.546*** | -0.232** |
| Total soil nitrogen (g kg^-1^) | -0.659*** | -0.728*** | -0.545*** | -0.232** |
| Total soil phosphorus (g kg^-1^) | -0.520*** | -0.523*** | -0.390*** | -0.005^NS^ |
| **Plants** |  |  |  |  |
| Aboveground net primary productivity (g m^-2^) | -0.648*** | -0.693*** | -0.538*** | -0.316*** |
| Plant species richness | -0.391*** | -0.504*** | -0.452*** | -0.244*** |
| Plant community carbon (%) | -0.717*** | -0.615*** | -0.620*** | -0.447*** |
| Plant community nitrogen (%) | 0.035^NS^ | 0.430*** | 0.504*** | 0.269*** |
| Plant community structure | 0.503*** | 0.553*** | 0.662*** | 0.547*** |
